# ICTV Virus Taxonomy Profile: *Ourmiavirus*

**DOI:** 10.1099/jgv.0.000725

**Published:** 2017-03-13

**Authors:** Massimo Turina, Brad I Hillman, Keramat Izadpanah, Mina Rastgou, Cristina Rosa, ICTV Report Consortium

**Affiliations:** ^1^​ Institute for Sustainable Plant Protection-CNR, Strada delle Cacce 73, Torino 10135, Italy; ^2^​ Department of Plant Biology and Pathology, Rutgers University, New Brunswick, NJ 08901, USA; ^3^​ Plant Virology Research Center, College of Agriculture, Shiraz University, Shiraz 71441 65186, Iran; ^4^​ Department of Plant Protection, College of Agriculture, Urmia University, Urmia 57561 51818, Iran; ^5^​ Department of Plant Pathology and Environmental Microbiology, College of Agricultural Sciences, The Pennsylvania State University, University Park, PA 16802, USA

**Keywords:** *Ourmiavirus*, ICTV Report, taxonomy

## Abstract

Members of the plant virus genus *Ourmiavirus* are characterized by having non-enveloped bacilliform virions with a series of discrete lengths from 30 to 62 nm composed of a single coat protein (CP). The genome consists of three positive-sense single-stranded RNAs, each encoding a single protein. The RNA-dependent RNA polymerase (RdRp) has closest similarity to that of viruses from the family *Narnaviridae*; the movement protein (MP) is similar to the MPs of tombusviruses; the CP shows limited similarity to the CPs of several plant and animal viruses. This is a summary of the International Committee on Taxonomy of Viruses (ICTV) Report on the taxonomy of the genus *Ourmiavirus*, which is available at www.ictv.global/report/ourmiavirus.

## Virion

**Fig. 1.**
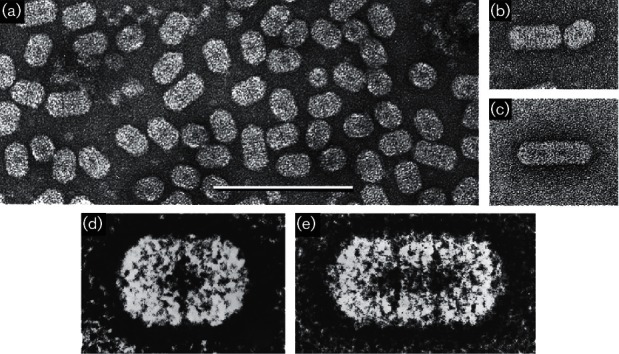
Virion morphology. (a–c) Negative-contrast electron micrographs (uranyl acetate) of purified particles of Ourmia melon virus. Bar, 100 nm. (d, e) Features of the two most common particle types, enhanced by photographic superimposition.

The bacilliform virions of Ourmiaviruses constitute a series of particles with conical ends (apparently hemi-icosahedra) and cylindrical bodies, 18 nm in diameter. The bodies of the particles are composed of a series of double discs, the most common particle having two discs (particle length 30 nm), a second common particle having three discs (particle length 37 nm), with rarer particles having four discs (particle length 45.5 nm) and six discs (particle length 62 nm). There is no envelope (Fig. 1 and Table 1).

**Table 1.** Characteristics of the genus *Ourmiavirus*

Typical member:	Ourmia melon virus-VE9 (RNA1: EU770623; RNA2: EU770624; RNA3: EU770625), species *Ourmia melon virus,* genus *Ourmiavirus*
Virion	Bacilliform (18×30–62 nm) with a single coat protein of 23.8 kDa
Genome	Tri-segmented positive-strand RNA (2.8; 1.1; 0.97 kb respectively)
Replication	Cytoplasmic; possible nucleolar localization of the coat protein; virion assembly coupled to active replication
Translation	From genomic uncapped RNAs; each genomic segment is monocistronic
Host range	Plants
Taxonomy	Unassigned genus; RdRp has similarities to recently discovered, unclassified invertebrate viruses related to members of the *Narnaviridae*

## Genome

**Fig. 2.**
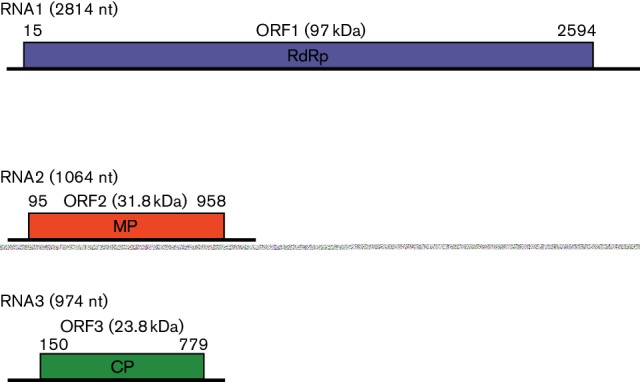
Genome organization. Diagram of the genome organization of Ourmia melon virus isolate VE9 showing the size of each RNA and the positions and sizes of the ORFs.

The genome is composed of three positive-sense single-stranded RNAs. In Ourmia melon virus, the RNAs are 2814, 1064 and 974 nt in length (Fig. 2) [1]. The single structural coat protein (CP; 23.8 kDa) is encoded by RNA3. The two non-structural proteins are the RNA-dependent RNA polymerase (RdRp; 97.5 kDa, encoded by RNA1) and the movement protein (MP; 31.6 kDa, encoded by RNA2) (Fig. 2). The sizes of genomic RNAs and predicted encoded proteins are similar for the other two species [1]. There is no evidence for the presence of subgenomic RNAs or for production of additional proteins by readthrough mechanisms.

## Replication

The putative RdRp carries the conserved GDD motif and has closest affinity with the RdRp of a number of viruses related to members of the family *Narnaviridae*, but is distinct from the RdRP of yeast viruses classified in the genus *Narnavirus* [2]. A protein fusion of the CP to GFP localizes specifically to the nucleolus [3] but there is no direct evidence of presence of the CP in the nucleus during infection [4]. Synthesis of CP from actively replicating RNA3 is necessary for both virion assembly and systemic infection of the host [5]. The MP may undergo post-translational modification. Alanine scanning mutagenesis of conserved residues in the MP showed its importance in determining symptoms, movement and formation of tubular structures that may play a role in cell-to-cell movement [6]. Details of replication are not known except that the CP interferes with the plant silencing defence only in the context of virus infection [4].

## Taxonomy

To date, three species of plant viruses are classified in the genus *Ourmiavirus: Ourmia melon virus*, members of which were initially isolated from melon plants in Iran [7], *Epirus cherry virus*, with members isolated from cherry trees in Greece [8], and *Cassava virus C*, members of which were isolated from cassava from equatorial Africa [9].

## Resources

Full ICTV Online (10th) Report: www.ictv.global/report/ourmiavirus.
